# Testosterone Decreases the Number of Implanting Embryos, Expression of Pinopode and L-selectin Ligand (MECA-79) in the Endometrium of Early Pregnant Rats

**DOI:** 10.3390/ijerph17072293

**Published:** 2020-03-29

**Authors:** Mohd Helmy Mokhtar, Nelli Giribabu, Naguib Salleh

**Affiliations:** 1 Department of Physiology, Faculty of Medicine, Universiti Kebangsaan Malaysia, Kuala Lumpur 56000, Malaysia; helmy@ukm.edu.my; 2 Department of Physiology, Faculty of Medicine, University of Malaya, Kuala Lumpur 50603, Malaysia; nelli.giribabu@um.edu.my

**Keywords:** testosterone, uterine receptivity, embryo implantation

## Abstract

Testosterone could have adverse effect on fertility. In this study, we hypothesized that this hormone could reduce the number of embryo implantations via affecting the normal endometrium ultrastructure and expression of endometrial proteins involved in implantation. Therefore, the aims were to identify these adverse testosterone effects. Methods: Intact pregnant rats were given 250 or 500 µg/kg/day testosterone for three days, beginning from day 1 of pregnancy. Rats were euthanized either at day 4 to analyze the ultra-structural changes in the endometrium and expression and distribution of MECA-79 protein, or at day 6 to determine the number of implantation sites. Results: Administration of 500 µg/kg/day testosterone suppresses endometrial pinopodes development and down-regulates expression and distribution of MECA-79 protein in the uterus. In addition, the number of implantation sites were markedly decreased. Conclusions: Changes in endometrial ultrastructure and expression of implantation protein in the endometrium in early pregnancy period could be the reason for failure of embryo implantation under testosterone influence.

## 1. Introduction

Implantation is a unique process that involves a series of physical and physiological interactions between embryo and the receptive endometrium [1]. Successful implantation requires transformation of the uterus into a receptive state [2]. Meanwhile, uterine receptivity is the state where the uterus is ready to accept the implanting embryo and its development is primarily controlled by ovarian steroids i.e., estrogen and progesterone [3]. Uterine receptivity is associated with the expression of specific molecular markers and characteristic changes to the uterus ultrastructure [2]. The specific uterus ultrastructural changes include the appearance of pinopodes, while specific molecules that are expressed exclusively during the uterine receptivity period include L-selectin ligand (MECA-79) [4]. 

Pinopodes are smooth, mushroom-like projections that arise from the apical surface of the receptive endometrium, and is considered as an essential microstructure marker of endometrial receptivity [5]. This projection has been reported to participate in embryo–endometrial interaction via L-selectin ligand [6], the latter of which consists of a carbohydrate epitope, MECA-79 [7]. Pinopodes are involved in pinocytosis of uterine fluid and macromolecules from the uterine lumen which facilitates luminal closure as well as apposition of blastocyst to the luminal epithelium [8].

In addition to estrogen and progesterone, testosterone has also been shown to affect the endometrium and embryo implantation. Testosterone has been found to disturb the regulation of fluid in the uterine lumen [9,10]. Additionally, this hormone has also been shown to disturb prostaglandin synthesis that is essential for implantation [11]. Limited findings indicate that testosterone could suppress the expression of HOXA-10, a signaling molecule that is necessary for the uterine receptivity development [12]. So far, studies on the effect of testosterone on embryo implantation and ultrastructural changes of the uterus during the peri-implantation period have not been identified. Therefore, the aims of this study were to investigate changes in the pinopode expression, expression of L-selectin ligand (MECA-79), as well as the overall effect on embryo implantation under testosterone influence. Our findings are important as they could help identify the mechanisms underlying adverse testosterone effect on embryo implantation. 

## 2. Materials and Methods

### 2.1. Animals & Hormone Treatment

In this study, adult female Sprague-Dawley (SD) rats that showed at least two consecutive, regular estrous cycles were used. Rats weighing 225 ± 25 g were caged overnight with male rats from the same species (1:1 ratio) and that had proven to be fertile. The next morning, successful copulation was confirmed by the presence of a vaginal plug. This day was designated as day 1 of pregnancy. The rats were caged under standard conditions (lights on 06:00 to 18:00 h; room temperature 24 °C; 4 animals per cage) and were fed with rat chow diet (Harlan, Germany) and tap water *ad libitum*. All experimental procedures were approved by University of Malaya Institutional Ethics Committee (2013-07-15/FIS/R/NS). 

Testosterone and peanut oil were obtained from Sigma–Aldrich, Saint Louis, MO, USA. Testosterone was dissolved in peanut oil and then injected subcutaneously behind the neck scruff in 0.1ml of volume. The groupings were as follows with n = 6 rats per group: 

Control—normal pregnant rats, (Group 1)

T250—pregnant rats treated with 250 µg/kg/day of testosterone (Group 2)

T500—pregnant rats treated with 500 µg/kg/day of testosterone (Group 3)

Groups 2 and 3 were treated with testosterone for three consecutive days starting from day 1 to day 3 of pregnancy. One cohort of rats from all groups was euthanized by cervical dislocation at day 4 and another cohort which also received testosterone from day 1 to day 3 was euthanized at day 6 of pregnancy. Uteri of the day 4 cohort were excised and subjected for morphological and molecular biological analyses. In the meantime, the number of implantation sites were determined for the cohort of rats that were sacrificed at day 6. 

### 2.2. Measurement of Serum Hormone Levels

Immediately following the euthanasia of pregnant rats at day 4, blood was collected via heart puncture, into a separator tube (SST) and was allowed to clot for 30 minutes at room temperature. The blood was centrifuged at 3000×g, for 15 min. Serum was aliquot and stored at −20 °C and hormone levels were determined by using enzyme-linked immunosorbent assay (ELISA) kit (CSB-E05110r-Estrogen, CSB-E07282r-Progesterone and CSB-E05100r-Testosterone,) (CUSABIO-USA), according to manufacturer guidelines. Absorbance was determined at a wavelength of 450 nm using a microplate reader (iMark; Bio-Rad, Hercules, CA, USA). 

### 2.3. Determination of the Number of Implantation Sites

Implantation sites were identified using Chicago Sky Blue dye (Sigma Aldrich, Dorset, UK) staining. The dye, dissolved in 0.85% sodium chloride, was injected intravenously to the rats under anesthesia. A total of 1 ml of dye was perfused through the tail vein. Chicago Sky Blue dye stained the areas of high vascularity, including the implantation sites. Images of the sites (blue-stained spots in the uterine horns) and inter-implantation sites (unstained spaces between two blue-stained bands) were captured. 

### 2.4. Transmission Electron Microscopy (TEM)

Following harvesting, uteri were immersed in 2.5% glutaraldehyde in 0.1M phosphate buffer, pH 7.4. A 2mm cross section of each uterine horn was cut and incubated overnight at 4 °C in 2.5% glutaraldehyde buffer. Following the removal of the buffer, samples were rinsed three times, 15 min each time, in 0.1 M phosphate buffer. Samples were then incubated in 1% osmium in 0.1 M phosphate buffer, rinsed and dehydrated in a series of ethanol (70–100%). Samples were incubated twice for 5 min in propylene oxide and then transferred to a rotor for 1 h at room temperature in 1:1 mixture of propylene oxide and epon (47% embed 812, 31% dodenyl succinic anhydride, 19% nadic methyl anhydride, 3% benzyldimethylamine) (Electron Microscopy Sciences, Hatfield, PA, USA). This was followed by overnight incubation in 1:2 propylene oxide–epon, and finally 100% epon for 2–3 h. Individual uterine tissues were embedded in 100% epon in silicon flat embedding molds, and capsules were polymerized at 60 °C for 48 h. Ultrathin transverse sections (70 nm) were prepared using a diamond knife (Diatome, Hatfield, PA, USA) on a MT 6000-XL ultramicrotome, captured on 300-mesh copper grids, and stained with 2% uranyl acetate.

### 2.5. Protein Distribution Analysis by Immunohistochemistry (IHC)

Uteri were fixed overnight in 4% paraformaldehyde (PFD) before being processed and dehydrated through increasing concentrations of ethanol, cleared in chloroform and blocked in a paraffin wax. Tissues were cut into 5 µm sections, deparaffinized in xylene, rehydrated in reducing concentrations of ethanol. Tri-sodium citrate (pH 6.0) was used for antigen retrieval, while 1% H_2_O_2_ in PBS was used to neutralize the endogenous peroxidase. Sections were blocked in 5% BSA for non-specific binding, prior to incubation with rat monoclonal primary antibody: MECA-79 (200 μg/0.1ml, 1:500) (Santa Cruz, SC 19602) in 5% BSA at room temperature for 1 h. After rinsing with PBS, sections were incubated with biotinylated secondary antibody for 30 min at room temperature and were then exposed to avidin and biotinylated HRP complex (Santa Cruz, California, USA) in PBS for another 30 min. The site of antibody binding was visualized by DAB (Diaminobenzidine HCl) (Santa Cruz, California, USA) which gave dark brown stains. Sections were counterstained with hematoxylin for nuclear staining. In this experiment, non-immune normal donkey serum was used as a negative control where no staining was observed. 

### 2.6. Protein Quantification by Western Blotting

Whole uterine tissues were snap frozen in liquid nitrogen and stored at −80 °C prior to protein extraction. Following protein extraction with PRO-PREP solution (Intron, South Korea), an equal amount of protein was mixed with a loading dye, boiled 5 min and separated with SDS-PAGE 12%. Protein was transferred onto a PVDF membrane (BIORAD, USA) and blocked with 5% BSA for 90 min at room temperature. The membrane was exposed to primary antibody, as above, at a dilution of 1:100 each in PBS containing 1% BSA and Tween-20 overnight. The blots were rinsed three times in PBS-T 5 min each. The membranes were then incubated with anti-goat and anti-rabbit horseradish peroxidase (HRP) conjugated secondary antibody (Santa Cruz Biotechnology, USA) at a dilution of 1:2000, for 1 h. The membranes were then subjected to Opti-4CN™ Substrate Kit (Bio-Rad, USA) to visualize the protein bands. Photos of the blots were captured using a gel documentation system and the density of each band was determined using Image J software (version 1.46j; National Institutes of Health, Bethesda, MD, USA). The ratio of each target band/GAPDH was calculated and was considered as the expression levels of the targets. The average ratio for each band was obtained from four different membranes representing four different animals receiving similar treatment. 

### 2.7. Statistical Analysis

One-way analysis of variance (ANOVA) was used to determine the levels of significance between the three groups. Values of *p* < 0.05 were considered as significant. In the meantime, post hoc statistical power analysis was performed, and all values were >0.8, indicating adequate sample size. 

## 3. Results

### 3.1. Plasma Levels of Estrogen, Progesterone and Testosterone in Pregnant Rats

Plasma level of estrogen at day 4 of pregnancy increased markedly following administration of testosterone (Table 1). The increase was dose-dependent. However, the plasma level of progesterone in the control group did not differ significantly from the testosterone-treated rats. Meanwhile, the plasma level of the testosterone increased with the increasing dose of subcutaneously administered testosterone. Following the administration of 500 µg/kg/day testosterone, the plasma level was approximately 50 folds higher than control.

### 3.2. Number of embryo implantation sites

**Figure 1:**
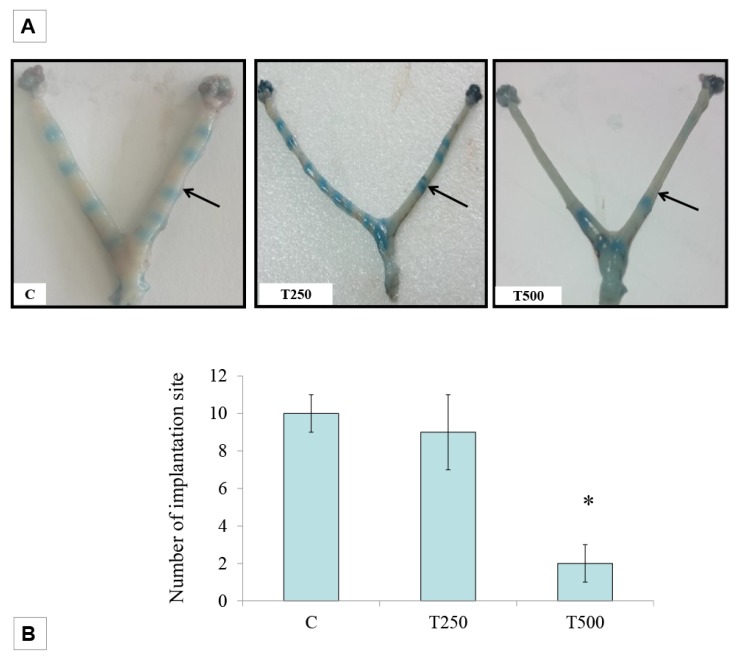
Implantation site at day 6 of pregnancy. (**A**) shows the image of the uterus in different treatment groups, with the blue band indicating the sites of implantation while (**B**) shows the analysis of the number of implantation sites in different treatment groups. C: normal pregnant rats (control), T250: 250 µg/kg/day testosterone and T500: 500 µg/kg/day testosterone, n = 6 per group.

The highest number of implantation sites could be seen in pregnant rats not receiving testosterone treatment (Figure 1). The number of implantation sites decreased significantly in rats receiving 500 µg/kg/day testosterone. In rats receiving 250 µg/kg/day testosterone, the number of implantation site was not significantly different when compared to control. 

### 3.3. TEM Images of Pinopodes

**Figure 2:**
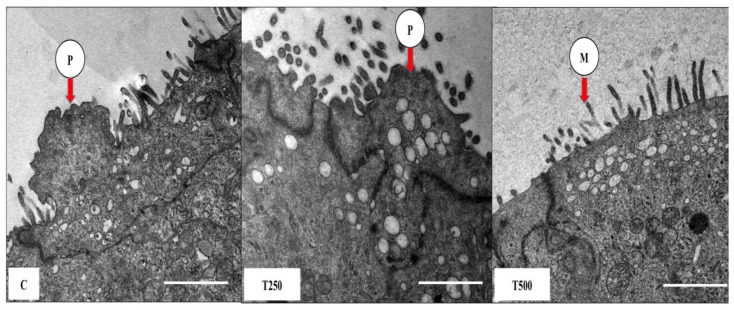
Representative transmission lecmicrograph (TEM) images of endometrium showing pinopodes and microvilli. Images were taken at day 4 of pregnancy following 3 days of T treatment. P: pinopode M: microvilli C: normal pregnant rats, T250: 250 µg/kg/day testosterone and T500: 500 µg/kg/day testosterone. Scale bar = 1 µm.

Pinopodes were seen in normal pregnant rats that did not receive testosterone treatment (Figure 2). Similarly, pinopodes could also be seen in rats receiving 250 µg/kg/rat testosterone. However, in rats that received 500 µg/kg/day testosterone, pinopodes were absent from the apical membrane, with only microvilli present. 

### 3.4. MECA-79 Protein Expression and Distribution

**Figure 3:**
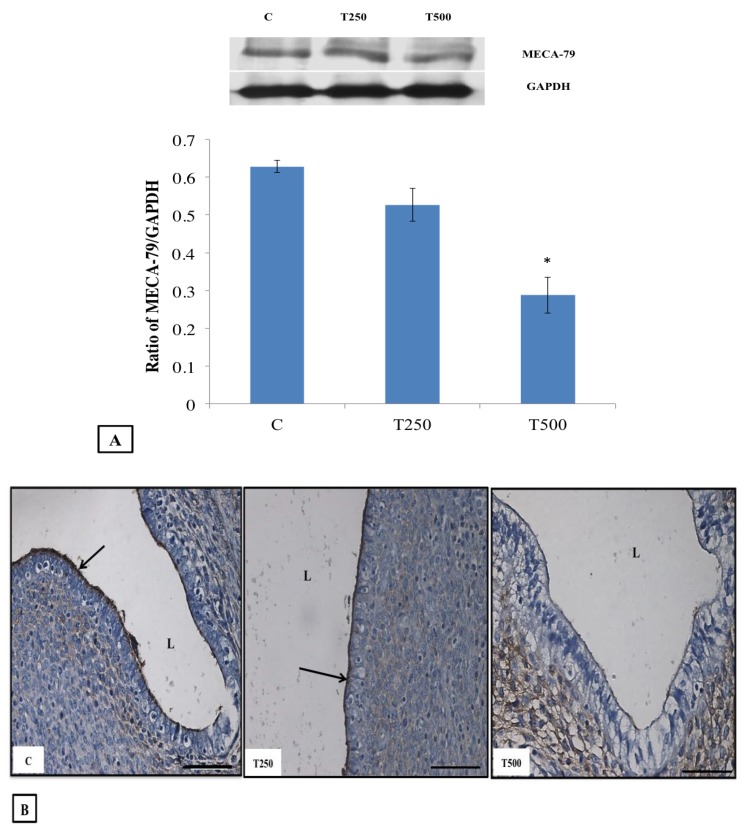
Expression and distribution of MECA-79 in the uterus: (**A**) shows the representative immunoblot image and analysis of the intensity of the MECA-79 protein band in different experimental groups while (**B**) shows the MECA-79 protein distribution at the apical membrane of the endometrial luminal epithelium in different experimental groups L: Lumen, C: normal pregnant rats, T250: 250 µg/kg/day testosterone-treated pregnant rats and T500: 500 µg/kg/day testosterone-treated pregnant rats. Arrows pointing towards the area of highest protein distribution (dark brown stain). Scale bar = 100 µm.

Figure 3A shows immunoblot image and analysis of intensity of MECA-79 protein band in the different experimental groups while Figure 3B shows the distribution of MECA-79 at the apical membrane of the endometrial luminal epithelia in different experimental groups. In this study, it was revealed that the highest MECA-79 protein expression and distribution could be seen in normal pregnant rats, followed by 250 µg/kg/day testosterone treated rats. However, administration of 500 µg/kg/day testosterone resulted in a significantly lower expression and distribution of MECA-79 in the endometrium.

## 4. Discussion

Embryo implantation involves a series of physical and chemical interactions between blastocyst and the receptive endometrium. This interaction occurs within a short period of time known as the implantation window period or endometrial receptivity period. During this period, the uterus is transformed into a receptive state [13]. Endometrial receptivity involves changes in the endometrium at various levels, including ultrastructural and molecular changes [14]. A number of molecular mediators, for example MECA-79, have been identified as being expressed exclusively during the uterine receptivity period [15]. Ultrastructural changes that appear exclusively within this period include the appearance of pinopodes, a microscopic protrusion from the apical membrane of the endometrial epithelium [16].

This study has shown that testosterone could adversely affect the development of endometrial receptivity, and ultimately, embryo implantation. Administration of exogenous testosterone, in particular, at a high dose of 500 µg/kg/day for three days beginning from the start of pregnancy, has been shown to reduce the expression of MECA-79 and suppress pinopode development, which could lead to a decrease in the number of implantation sites in the uterus. These findings indicate that exposure to a high dose of testosterone could potentially decrease fertility. A smaller effect was observed at lower testosterone doses, whereby no significant changes to these parameters were observed, indicating that at this dose, testosterone might not be harmful to embryo implantation. There is a possibility that at this lower dose, most testosterone molecules might undergo aromatization to estrogen [17], an event that has been reported to occur in ovary and adipose tissue [18]. Thus, this would result in a smaller rise in serum testosterone level (five-fold) as compared to thirty-fold rise in serum testosterone levels, following administration of a high dose of testosterone. In the latter, the presence of testosterone at a high dose could saturate the aromatase enzyme, causing a high amount of unconverted testosterone to remain in circulation. In parallel to these changes, it could be seen that estrogen levels rise drastically following administration of 250 µg/kg/day testosterone (two fold higher than control), however, the levels rise only 2.5-fold higher than the control following the administration of 500 µg/kg/day testosterone, the latter of which might be caused by over-saturation of the aromatase. 

Our findings showed that testosterone at high dose (500 µg/kg/day) almost completely suppressed pinopode expression. Consequently, attachment of the embryo to the receptive endometrium might be impaired. The attachment phase starts once the blastocyst enters the uterine cavity [19]. It was postulated that this phase is initiated by the removal of fluid from the uterine cavity, which brings the two opposing uterine walls into contact with one another, sandwiching the blastocyst [20]. This event is shown to be under the control of progesterone [21] and might involve the pinopodes, the appearance of which is progesterone dependent [22] and in rats have been shown to perform pinocytosis [23]. Therefore, there is a possibility that in the presence of testosterone, particularly at high doses, the absence or under appearance of pinopodes could interfere with fluid removal from the uterine lumen. 

Apart from the role that they play in uterine fluid removal, pinopodes have also been postulated to be able to initiate the adhesion phase of implantation, due to the fact that they contain L-selectin [24]. A high expression of L-selectin and its ligand, MECA-79, during the embryo implantation period has been proposed to help increase the stickiness of the endometrial epithelium toward the implanting blastocyst [25]. The sticky surface will anchor the blastocyst firmly to the endometrial wall for the invasion phase to start [26]. As the expression of MECA-79 in the uterus was markedly reduced following the administration of a high dose of testosterone, the adhesion process could well have been compromised, which ultimately would interfere with successful embryo implantation. It is highly possible that the significantly reduced number of implantation sites observed might be caused by the reduced levels of MECA-79, as adequate expression levels of this molecule in humans correlate with higher levels of fertility, and reduced expression could result in infertility. 

## 5. Conclusions

In conclusion, disturbances in uterine ultrastructure i.e., reduced appearance of pinopodes as well as decreased expression and distribution of implantation molecules such as MECA-79, which is associated with pinopodes, might decrease fertility, as observed in rats, following the administration of a high dose of testosterone. These findings could be extended to humans, where subfertility or infertility related to high plasma testosterone examples in such diseases as polycystic ovarian disease (PCO), or following excessive anabolic steroid intake, might be caused by similar mechanisms that interfere with normal uterine function during the implantation window period. 

## Figures and Tables

**Table 1:** Plasma level of reproductive hormones at day 4 of pregnancy. * *p* < 0.05 compared to C. C: control, T250: 250 µg/kg/day testosterone and T500: 500 µg/kg/day testosterone.

HORMONES	GROUPS	PLASMA LEVELS
ESTROGEN	C	49.65 ± 3.66 pmol/L
	T250	*83.35 ± 2.91 pmol/L
	T500	*102.67 ± 1.76 pmol/L
PROGESTERONE	C	166.93 ± 8.71 nmol/L
	T250	134.8 ± 12.20 nmol/L
	T500	161.73 ± 3.98 nmol/L
TESTOSTERONE	C	0.60 ± 0.2 nmol/L
	T250	*5.90 ± 1.44 nmol/L
	T500	*30.07 ± 1.47 nmol/L
